# Childhood trauma and aggression in incarcerated males: the parallel mediating effects of empathic concern and perspective-taking

**DOI:** 10.3389/fpsyg.2026.1806562

**Published:** 2026-06-24

**Authors:** Guiqin Liu, Yongzhi Jiang, Xiaoli Bai, Qing Liang, Zuoyi Wang, Min Li

**Affiliations:** 1 School of Education Science, Inner Mongolia Minzu University, Tongliao, China; 2 Inner Mongolia Student Bullying Prevention Research Center, Tongliao, China; 3 Tongliao Prison, Tongliao, China

**Keywords:** aggression, childhood trauma, empathic concern, incarcerated males, perspective-taking

## Abstract

Childhood trauma is a critical and highly destructive risk factor for aggression. To examine the relationship between childhood trauma and aggression in incarcerated males, this study recruited 739 incarcerated males from Inner Mongolia, China. The Childhood Trauma Questionnaire (CTQ), the Interpersonal Reactivity Index (IRI), and the Buss-Perry Aggression Questionnaire (BPAQ) were used to measure childhood trauma, empathic concern and perspective-taking, and aggression, respectively. This study aimed to examine whether empathic concern and perspective-taking have parallel mediating effects in the relationship between childhood trauma and aggression. The results showed that: (1) Childhood trauma was significantly positively correlated with aggression (*r* = 0.303, *p* < 0.001), and significantly negatively correlated with both perspective-taking(*r* = −0.253, *p* < 0.001) and empathic concern(*r* = −0.367, *p* < 0.001); aggression was significantly negatively correlated with both perspective-taking(*r* = −0.087, *p* < 0.05) and empathic concern(*r* = −0.275, *p* < 0.001). (2) Regression-based mediation analysis indicated a positive association between childhood trauma and aggression (total effect value β = 0.301, *p* < 0.001). Empathic concern partially mediated this association (indirect effect value β = 0.076, accounting for 25.25% of the total effect value). The indirect effect of perspective-taking was not significant. Drawing on the General Aggression Model and social learning theory, this study found a positive association between childhood trauma and aggression, and this association could be partially explained by empathic concern. The findings enhance the understanding of the complex relationship between them and have implications for intervention practices with incarcerated individuals.

## 1 Introduction

Aggression, as a prominent externalizing behavior problem in humans, has become a focus of widespread concern across multiple disciplines in society. Aggressive behavior not only causes harm to individuals and others but also has severe consequences for families and society (Salimi et al., 2019). An individual’s aggression is influenced by both environmental and personal factors, among which early adverse growth experiences, especially childhood trauma, have been recognized as a critical and highly destructive risk factor for aggression (Moore et al., 2020). A meta-analysis indicated that approximately half of incarcerated individuals in Canadian prisons had experienced abuse during childhood (Bodkin et al., 2019). Therefore, exploring the association pathways and potential psychological processes between childhood trauma and aggression is of significant reference value for preventing violence at its source and inspiring scientific interventions.

### 1.1 The association between childhood trauma and aggression

The World Health Organization (WHO) defines child maltreatment as all types of abuse, neglect, or other exploitation by parents or caregivers that result in actual or potential harm to the child’s health integrity (World Health Organization, 2006). In the present study, the concept of childhood trauma is adopted, and it is operationalized according to the Childhood Trauma Questionnaire (CTQ) developed by Bernstein et al. (2003), which includes three types of abuse (emotional abuse, physical abuse, and sexual abuse) and two types of neglect (emotional neglect and physical neglect). Childhood maltreatment is closely associated with aggressive behavior in adulthood, including the perpetration of intimate partner violence, child abuse, and violent crime (Wekerle et al., 2001). A number of studies have shown a significant positive association between childhood maltreatment and aggressive behavior (Sun et al., 2017; Odacı and Çelik, 2020; Li et al., 2022; Ran et al., 2023; Zou et al., 2024; Tian et al., 2025). Research on juvenile offenders and adult incarcerated individuals indicates that childhood maltreatment directly and positively predicts aggression (Ma et al., 2020; Xie et al., 2020; Ma et al., 2022; Wu et al., 2023). Lansford et al. (2007) found that even after controlling for multiple variables such as socioeconomic status, children who had experienced physical abuse had significantly more court records of violent crime compared to their non-abused peers. Mersky and Topitzes (2010) studied 1,539 ethnic minority children from economically disadvantaged families; these children were assessed in preschool and followed up to approximately age 24. The results showed that childhood trauma experience was a significant predictor of later arrest (Mersky and Topitzes, 2010). Another study found that among 196 violent offenders, 137 had experienced childhood abuse and neglect (Widom, 2014). The above studies consistently indicate that childhood trauma and aggression are closely associated.

Individuals who experienced maltreatment during childhood may be at higher risk of perpetrating violence or engaging in illegal behavior in adulthood, a phenomenon referred to as the “cycle of violence” or the “intergenerational transmission of abuse” (Widom, 2014). Regarding the association pathways between childhood trauma and aggression, social learning theory suggests that aggressive behavior can be learned through observation and imitation. In a violent childhood environment, children may observe and imitate the behavior of their abusers and internalize aggression as a behavioral pattern for solving problems (Akers et al., 1995; Yoon et al., 2017). Accordingly, this study proposes Hypothesis 1: There is a positive association between childhood trauma and aggression in incarcerated males.

### 1.2 Hypotheses on the mediating effects of empathic concern and perspective-taking

In addition to examining the direct association between childhood trauma and aggression, this study also aimed to examine whether childhood trauma is indirectly associated with aggression through the mediating effect of empathy. Empathy refers to the ability to feel and understand another person’s emotions from their frame of reference (American Psychological Association [APA], 2023). This ability can be measured from a multidimensional perspective, encompassing both affective and cognitive dimensions. Among these, empathic concern refers to other-oriented feelings of sympathy and concern in response to others’ misfortune, belonging to affective empathy. Perspective-taking refers to the tendency to spontaneously adopt another person’s point of view, belonging to cognitive empathy (Davis, 1983).

The General Aggression Model (GAM) provides an integrative theoretical framework for understanding the generation of aggressive behavior. The model posits that specific input variables (e.g., personal experiences) influence an individual’s internal states (cognition, affect, and arousal), which in turn affect judgment and decision-making processes, ultimately influencing aggression (Anderson and Bushman, 2002). Based on this framework, the present study operationalizes childhood trauma as an input variable, positions aggression as the outcome, and focuses on empathic concern and perspective-taking—representing the affective and cognitive components of internal states, respectively—to examine their mediating roles in the input-outcome relationship.

Empirical studies have provided preliminary support for the various links specified in the GAM pathway described above. First, childhood trauma (input variable) is associated with empathy (internal state). Meta-analytic findings indicate a significant negative correlation between childhood trauma and empathy (Meng et al., 2023; Zhang H. P. et al., 2023). A study on individuals in compulsory drug rehabilitation also demonstrated a significant negative correlation between childhood trauma and empathy (Ren et al., 2018). Second, empathy (internal state) is associated with aggression (outcome). Research has shown that both empathic concern and perspective-taking are significantly negatively correlated with aggressive behavior (Song et al., 2025; Zhang, 2021). Taken together, these findings point to the possibility that empathic concern and perspective-taking may play a mediating role in the relationship between childhood trauma and aggression. Accordingly, we propose Hypothesis 2: Empathic concern and perspective-taking have significant mediating effects in the association between childhood trauma and aggression.

According to social learning theory, childhood trauma is directly associated with aggression in incarcerated individuals, without necessarily relying on proximal situational factors. In contrast, the GAM suggests that childhood trauma, as a distal factor, may be indirectly associated with aggression through a proximal factor such as empathy. At present, empirical evidence supporting these theoretical perspectives remains limited, and the complex pathways underlying the cycle of violence remain largely unknown. This study aims to contribute to the existing literature by constructing an integrated “childhood trauma–empathy–aggression” mediation model to examine the cycle of violence. Incarcerated males were selected as the target sample because this population exhibits typical manifestations of aggression; examining the psychological pathways of aggression in this group can maximally reflect the strength of the association between childhood trauma and adulthood aggression. Furthermore, empathy training is a routine component of psychological correction programs in correctional facilities both domestically and internationally. If the mediation analyses in this study reveal significant indirect effects of empathic concern and perspective-taking in the association between childhood trauma and aggression, the findings will provide preliminary theoretical insights for psychological correction work with incarcerated populations and may hold certain practical reference value.

## 2 Research methods

### 2.1 Research subjects

In May 2025, commissioned by the Education Section of Tongliao Prison in Inner Mongolia Autonomous Region, China, we conducted a mental health assessment of male incarcerated individuals and provided 1-year psychological counseling services. Prior to the project, the researchers submitted a research application and ethics approval documents to the prison administration. After obtaining approval, prison officers screened eligible individuals from the prison’s integrated management system according to the inclusion and exclusion criteria. This study ultimately employed a convenience sampling method to select male incarcerated individuals with normal intelligence, literacy skills, and no mental illness as participants. Two prison officers and two researchers served as test administrators. After explaining the purpose of the survey, providing confidentiality assurances, and obtaining informed consent, group testing was conducted inside the prison. Both the prison officers and the researchers received pre-test training to ensure standardization of the data collection process. Data collection and data entry were completed between May and June 2025. A total of 800 questionnaires were distributed, and 739 valid questionnaires were obtained, yielding an effective response rate of 92.38%.

The final sample consisted of 739 valid participants, with an age range of 23–55 years (*M* = 39.96, SD = 7.90). The age distribution was as follows: 23–33 years, 21.80%; 34–44 years, 44.10%; 45–55 years, 34.10%. Regarding marital status, 108 participants (14.6%) were unmarried, 504 (68.2%) were married, 112 (15.2%) were divorced, and 15 (2%) were widowed.

### 2.2 Inclusion and exclusion criteria

#### 2.2.1 Inclusion criteria

(1). Incarcerated males aged 18–60;
(2). Normal intelligence;
(3). Able to understand written Chinese and have the cognitive ability to complete the questionnaire.

#### 2.2.2 Exclusion criteria

(1). Currently in a strict supervision period, confinement period, or isolation review period;
(2). Diagnosed by the prison’s psychological correction center as having severe mental disorders such as schizophrenia, bipolar disorder, or currently in an acute drug/alcohol withdrawal period;
(3). Experiencing severe violent impulses and not suitable for contact with external researchers;
(4). Illiterate or unable to complete the questionnaire due to visual or hand injuries.

### 2.3 Research tools

#### 2.3.1 Childhood Trauma Questionnaire (CTQ)

This questionnaire was developed by Bernstein et al. (2003) and revised by Zhao et al. (2005). It consists of 25 measurement items and three validity items, requiring respondents to recall their experiences before the age of 16. The questionnaire is divided into five dimensions: physical abuse (PA), emotional abuse (EA), sexual abuse (SA), physical neglect (PN), and emotional neglect (EN), and uses Likert five-point scoring. The higher the questionnaire score, the more childhood trauma experiences the individual has. A study examined the applicability of the CTQ in a sample of incarcerated individuals from a prison in Beijing, and the results showed that the CTQ had good internal consistency (Cronbach’s α = 0.83) in the incarcerated population (Xie et al., 2020). In the present study, Cronbach’s alpha coefficient for the total scale was 0.854, and McDonald’s ω coefficient was 0.890. The results of the confirmatory factor analysis indicated that the model fit was acceptable. χ^2^ = 1060.538, df = 265, χ^2^/df = 4.002, RMSEA = 0.050, CFI = 0.869, TLI = 0.852.

#### 2.3.2 Interpersonal reactivity index (IRI)

This scale was developed by Davis (1983) and the Chinese version revised by Zhang et al. (2010). Two subscales, Perspective-Taking (PT) and Empathic Concern (EC), were used. The PT subscale assesses the tendency to spontaneously adopt another person’s point of view (cognitive empathy), while the EC subscale assesses the tendency to experience sympathy and concern for others’ misfortune (affective empathy). Both subscales were rated on a five-point Likert scale, with higher scores indicating higher levels of perspective-taking and empathic concern. The IRI has shown good internal consistency in adolescents (Cronbach’s α = 0.94) (Zhang Y. Y. et al., 2023). In the present study, Cronbach’s alpha coefficients for PT and EC were 0.767 and 0.774, respectively. McDonald’s omega coefficients for PT and EC were 0.772 and 0.788, respectively. The results of the confirmatory factor analysis indicated that the model fit was acceptable. χ^2^ = 140.279, df = 43, χ^2^/df = 3.262, RMSEA = 0.055, CFI = 0.921, TLI = 0.878.

#### 2.3.3 Buss-perry aggression questionnaire (BPAQ)

The aggression questionnaire was adopted, which was originally developed by Buss and Perry (1992) and revised by Luo (2008). It consists of four dimensions: physical aggression, verbal aggression, anger, and hostility, with a total of 29 items. The questionnaire uses Likert five-point scoring. The higher the score, the stronger the aggression. A study examined the applicability of the CTQ in a sample of incarcerated individuals, and the results showed that the CTQ had good internal consistency (Cronbach’s α = 0.94) in the incarcerated population (Xie et al., 2020). In the present study, Cronbach’s alpha coefficient for the total scale was 0.850, and McDonald’s omega coefficient was 0.867. The results of the confirmatory factor analysis indicated that the model fit was acceptable. χ^2^ = 922.394, df = 344, χ^2^/df = 2.681, RMSEA = 0.047, CFI = 0.900, TLI = 0.881.

### 2.4 Procedures

This study adopted a combined approach of commissioned recruitment and voluntary registration. Prison officers responsible for psychological work in each unit verbally announced, at the end of routine educational activities, that “a psychological study on childhood experiences is being conducted; participation is voluntary and will not affect your evaluation or sentence reduction.” Officers distributed informed consent forms to interested incarcerated individuals. Researchers then held a unified information session in the presence of but without the participation of prison officers. Incarcerated individuals willing to participate signed up with their unit officer within a designated period and signed an informed consent form. Researchers enrolled participants in the order of registration.

Given that the participants were incarcerated individuals, the informed consent procedure specifically strengthened the safeguards of “voluntariness” and “confidentiality.” The specific process was as follows:

(1). Researchers commissioned prison officers to distribute the informed consent form to potential participants 24 h in advance. Prior to formal testing, researchers highlighted three key points: “study participation is entirely unrelated to incarceration evaluations,” “refusal or withdrawal will not result in any adverse consequences,” and “all responses, except in cases of extreme security risks, will be kept strictly confidential.” Incarcerated individuals were informed that the questionnaire content would be used solely for psychological counseling and research and that the survey process would remain strictly anonymous.
(2). After thorough consideration, participants voluntarily signed the written informed consent form. Researchers confirmed participants’ understanding by asking questions regarding the core clauses of the informed consent form.
(3). In anticipation of potential emotional reactions triggered by the Childhood Trauma Questionnaire, a psychological counselor was available throughout the study to provide immediate support, and mental health assistance channels were listed in the informed consent form.
(4). Participants were informed that if they felt discomfort during the completion of the questionnaire, they had the right to withdraw unconditionally at any stage prior to the completion of data collection, and any completed responses would be destroyed on the spot.

### 2.5 Data analysis

Data preprocessing, descriptive statistics, reliability analysis, analysis of variance (ANOVA), and correlation analyses among variables were conducted using SPSS 20.0. McDonald’s omega coefficients for the measurement instruments were calculated using Jamovi software. Confirmatory factor analysis (CFA) was performed using AMOS 24.0. Heterogeneity analysis and forest plots were generated using GraphPad Prism. The PROCESS 3.0 macro program was used for the mediation model test, and Model 4 provided by Hayes was selected for analysis. The bias-corrected non-parametric percentile Bootstrap method (95% confidence interval) was used to test the significance level of the mediation effect. Statistically, if the 95% confidence interval does not include zero, it is considered that the indirect effect is significant.

## 3 Results

### 3.1 Common method bias test

The Harman single-factor method was used to test for common method bias (Zhou and Long, 2004). The results showed that there were 17 factors with eigenvalues greater than 1, and the variance explained by the first common factor was 15.77%, which was less than the critical standard of 40%. Therefore, there was no significant problem of common method bias in this study.

### 3.2 Descriptive statistics and correlation analysis

Table 1 presents the means, standard deviations, and correlation coefficients for the various variables. Childhood trauma was significantly positively correlated with aggression (*r* = 0.303, *p* < 0.001), and was significantly negatively correlated with perspective-taking (*r* = −0.253, *p* < 0.001) and empathic concern (*r* = −0.367, *p* < 0.001); aggression was also significantly negatively correlated with perspective-taking (*r* = −0.087, *p* < 0.05) and empathic concern (*r* = −0.275, *p* < 0.001). Except for verbal aggression, which was not significantly correlated with childhood trauma, physical neglect and emotional neglect, aggression and its dimensions were significantly positively correlated with each dimension of childhood trauma. The research results revealed that there was a positive correlation ranging from small to large between aggression and each dimension of childhood trauma, with the correlation coefficient r ranging from 0.178 (aggression and physical neglect) to 0.772 (aggression and sexual abuse). The specific correlation situations among the variable dimensions are shown in Table 1.

**TABLE 1:** Descriptive statistics and correlation matrix of main variables (*N* = 739).

Variable	*M*	SD	1	2	3	4	5	6	7	8	9	10	11	12	13
1 Childhood trauma	51.14	17.02	1.00	–	–	–	–	–	–	–	–	–	–	–	–
2 Physical neglect	10.20	2.78	0.631***	1.00	–	–	–	–	–	–	–	–	–	–	–
3 Emotional neglect	10.83	5.72	0.725***	0.342***	1.00	–	–	–	–	–	–	–	–	–	–
4 Sexual abuse	12.83	4.20	0.318***	0.174***	0.156***	1.00	–	–	–	–	–	–	–	–	–
5 Physical abuse	13.39	4.67	0.220***	0.158***	0.102**	0.460***	1.00	–	–	–	–	–	–	–	–
6 Emotional abuse	7.79	4.07	0.760***	0.423***	0.345***	0.287***	0.158***	1.00	–	–	–	–	–	–	–
7 Perspective-taking	17.54	4.03	−0.253***	−0.192***	−0.267***	−0.060	−0.062	−0.120**	1.00	–	–	–	–	–	–
8 Empathic concern	23.11	4.43	−0.367***	−0.248***	−0.278***	−0.248***	−0.180***	−0.285***	0.380***	1.00	–	–	–	–	–
9 Aggression	76.98	19.08	0.303***	0.178***	0.140***	0.772***	0.702***	0.272***	−0.087*	−0.275***	1.00	–	–	–	–
10 Physical aggression	22.06	8.15	0.270***	0.158***	0.135***	0.558***	0.585***	0.257***	−0.134***	−0.267***	0.882***	1.00	–	–	–
11 Verbal aggression	15.36	2.85	0.072	0.005	−0.011	0.510***	0.144***	0.128***	0.148***	−0.058	0.380***	0.171***	1.00	–	–
12 Anger	17.76	6.64	0.298***	0.197***	0.155***	0.662***	0.668***	0.226***	−0.133	−0.234***	0.858***	0.706***	0.198***	1.00	–
13 Hostility	21.81	6.46	0.215***	0.123***	0.088***	0.710***	0.584***	0.189***	−0.018	−0.208***	0.791***	0.541***	0.260***	0.525***	1.00

*P < 0.05, **P < 0.01, ***P < 0.001.

### 3.3 Parallel mediation analysis of perspective-taking and empathic concern

To determine the covariates to be included in the mediation model, this study examined the associations between demographic variables and aggression using Pearson correlation analysis and one-way analysis of variance (ANOVA). The results showed that neither age [*r* = −0.025, *p* = 0.491; F(3, 735) = 2.473, *p* = 0.085] nor marital status [F(3, 735) = 1.677, *p* = 0.170] was significantly associated with aggression (*p* > 0.05). Therefore, these variables were not included as covariates in the subsequent parallel mediation analysis.

All variables were standardized prior to the analyses, and Model 4 of the PROCESS v3.0 macro was used to test the parallel mediation model of perspective-taking and empathic concern in the association between childhood trauma and aggression among incarcerated individuals. The regression results indicated that childhood trauma had a significant positive association with aggression (β = 0.301, *p* < 0.001), a significant negative association with perspective-taking (β = −0.230, *p* < 0.001), and a significant negative association with empathic concern (β = −0.368, *p* < 0.001). When childhood trauma, perspective-taking, and empathic concern were simultaneously entered into the model with aggression as the outcome, childhood trauma remained significantly and positively associated with aggression (β = 0.239, *p* < 0.001), perspective-taking was not significantly associated with aggression (β = 0.057, *p* > 0.05), and empathic concern had a significant negative association with aggression (β = −0.206, *p* < 0.001). Specific results are shown in Table 2.

**TABLE 2:** Results of regression analyses for the parallel mediation model.

Regression equation		Overall fitting index			Regression coefficient significance		Confidence interval
Independent variable	Dependent variable	R	R^2^	F	β	*t*	95% CI
Aggression	Childhood trauma	0.302	0.091	73.692***	0.301	8.584***	[0.232, 0.370]
Perspective-taking	Childhood trauma	0.253	0.064	50.190***	−0.230	−7.085***	[−0.294, −0.166]
Empathic concern	Childhood trauma	0.367	0.135	114.699***	−0.368	−10.710***	[−0.435, −0.300]
Aggression	Childhood trauma	0.353	0.124	34.713***	0.239	6.377***	[0.165, 0.312]
	Perspective-taking				0.057	1.371	[−0.025, 0.138]
	Empathic concern				−0.206	−5.268***	[−0.283, 0.129]

***P < 0.001.

**FIGURE 1:**
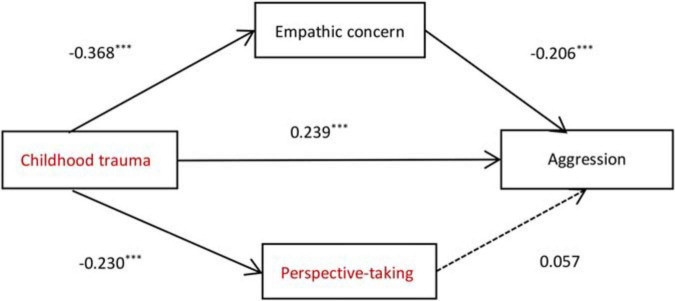
Empathic concern and perspective-taking as mediators in the association between childhood trauma and aggression. ***p < 0.001.

Further mediation analysis indicated that the total association between childhood trauma and aggression was significant (β = 0.301, SE = 0.035, *p* < 0.001), bootstrap 95% CI [0.232, 0.370], and the direct association between the two variables was also significant (β = 0.239, SE = 0.037, *p* < 0.001), bootstrap 95% CI [0.165, 0.312]. Empathic concern as a mediator showed a significant indirect effect of 0.076 (SE = 0.015), accounting for 25.25% of the total effect, bootstrap 95% CI [0.048, 0.110] (excluding zero), indicating that the mediation effect was significant. Perspective-taking as a mediator showed an indirect effect of −0.013 (SE = 0.011), bootstrap 95% CI [−0.034, 0.009](including zero), indicating that the mediation effect was not significant. Specific results are shown in Figure 1 and Table 3.

**TABLE 3:** Direct and indirect effects between latent variables.

Independent variable	Effect type	Path	Efficiency value	SE	Relative effect value	95% CI
Childhood trauma	Direct effect (β)	Childhood trauma → aggression	0.239	0.037	79.40%	[0.165, 0.312]
	Indirect effect (β)	Childhood trauma → perspective-taking → aggression	−0.013	0.011	−4.32%	[−0.034, 0.009]
		Childhood trauma → empathic concern → aggression	0.076	0.015	25.25%	[0.048, 0.110]
	Total effect (β)	–	0.301	0.035	100%	[0.232, 0.370]

### 3.4 Heterogeneity analysis

**FIGURE 2:**
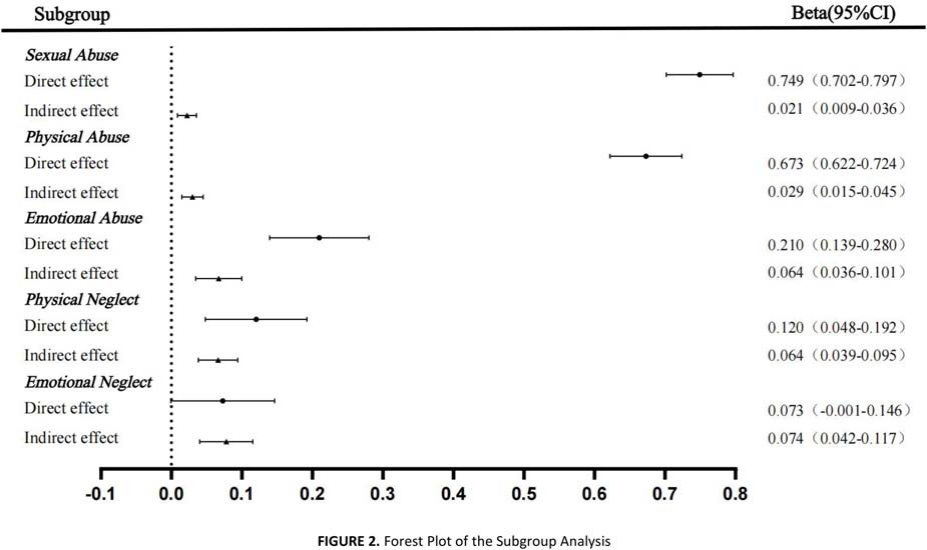
Forest plot of the subgroup analysis. The direct effect is childhood trauma → aggression, the indirect effect is childhood trauma → empathic concern → aggression.

As illustrated in the mediation model in Figure 1, the results presented in the forest plot in Figure 2 reveal the differences across various types of childhood trauma on each path. The present study found that the direct association between emotional neglect and aggression was not significant, whereas the direct associations between the other four dimensions (sexual abuse, physical abuse, emotional abuse, and physical neglect) and aggression were all significant. This finding suggests that the association pattern between emotional neglect and aggression may differ from that of other types of trauma. The direct effect of sexual abuse on aggression was the most significant (β = 0.749, Bootstrap 95% CI [0.702, 0.797]), while the direct effect of emotional neglect was the smallest (β = 0.073, Bootstrap 95% CI [−0.001, 0.146]). In contrast, the indirect effects of sexual abuse and empathy attention on aggression were the smallest (β = 0.021), while those of emotional neglect and empathy attention on aggression were the largest (β = 0.074). Sexual abuse and physical abuse showed completely non-overlapping confidence intervals with those of the other three dimensions, suggesting that the effects are qualitatively different. Specific results are shown in Figure 2.

## 4 Discussion

### 4.1 Correlation between childhood trauma, aggression, perspective-taking, and empathic concern

Firstly, this study found that childhood trauma was significantly positively correlated with the level of aggression among incarcerated individuals. This result is consistent with the view of of social learning theory, which suggests that children growing up in an abusive environment may view aggressive behavior as an effective way of interacting or solving problems and imitate and internalize it (Akers et al., 1995). The study also found that the correlation coefficients between aggression and various dimensions of childhood trauma ranged from small to large: emotional neglect (*r* = 0.140), physical neglect (*r* = 0.178), emotional abuse (*r* = 0.272), sexual abuse (*r* = 0.702), and physical abuse (*r* = 0.772). This indicates that among the various dimensions of childhood trauma, physical abuse and sexual abuse have the strongest relationship with aggression. As the Dimensional Model of Adversity and Psychopathology (DMAP) emphasizes, different types of abuse have differential impacts on individuals, and this diversity is crucial when considering the association between childhood trauma and aggressive behavior (McLaughlin and Sheridan, 2016). The results of this study indicate that the more directly violent or traumatic the type of childhood trauma (such as physical abuse and sexual abuse) involves, the stronger the correlation between the childhood trauma and aggression. This may be because physical abuse and sexual abuse may involve caregivers demonstrating aggressive behavior through explicit and relational ways, thereby increasing the risk of aggressive behavior in individuals (Ford et al., 2012; Widom, 2014). This also aligns with the Social Learning Theory, which suggests that children may internalize and repeat aggressive behavior by observing and experiencing violence. The correlation between neglect (emotional neglect and physical neglect) and aggression is relatively weak, indicating that neglect is more likely to lead to passive or introverted problems rather than active aggression.

Secondly, this study found that childhood trauma was significantly negatively correlated with perspective-taking and empathic concern. This is consistent with existing research (Almeida et al., 2025). This can be understood from the perspective of attachment theory: a childhood environment filled with abuse or neglect may hinder the development of secure attachment, and insecure attachment may further impair the individual’s ability to understand, recognize, and differentiate the emotions of others, i.e., empathic impairment (Yoo et al., 2013).

Finally, this study found that both perspective taking and empathic concern were significantly negatively correlated with aggression, which is consistent with our expectations. The empathy-altruism hypothesis suggests that the capacity to deeply understand the feelings and situations of others activates prosocial motivation in individuals and inhibits their tendency to harm others for personal gain. When individuals can “empathically feel” the pain of others, their likelihood of engaging in aggressive behavior naturally decreases (Batson and Shaw, 1991; Batson et al., 1997). Furthermore, individuals with lower levels of empathy tend to have weaker feelings of shame and guilt; when faced with conflict situations, they may be less concerned about the harm their actions cause to others, thereby exhibiting higher levels of aggression (Trivedi-Bateman and Crook, 2021).

### 4.2 Mediating effects of empathic concern and perspective taking between childhood trauma and aggression

#### 4.2.1 Analysis and discussion of the mediating effect of empathic concern

The mediation analysis showed that empathic concern played a partial mediating role in the association between childhood trauma and aggression. This finding suggests that the covariance between childhood trauma and aggression can be decomposed into two parts: a direct association between childhood trauma and aggression, and an indirect association formed through empathic concern.

First, there is a direct association between childhood trauma and aggression among male incarcerated individuals. This finding is consistent with previous studies (Ma et al., 2022; Xie et al., 2020). It aligns with Hypothesis H1 and also with the core expectations of social learning theory. According to this theory, abused children observe parents using aggressive and violent behaviors, thereby coming to view aggression as an acceptable way to deal with anger and punishment, while failing to learn other acceptable coping methods for handling negative emotions (Calvete and Orue, 2012). Through long-term observation, children store this “aggression–control–problem-solving” interaction pattern as a preferred script for problem-solving. When they encounter similar conflicts in situations outside the family, they activate and imitate this internalized aggressive script, thus exhibiting higher levels of aggression. The findings from the incarcerated sample in this study provide new empirical support for this theory, indicating that the covariance between childhood trauma and adult aggression may partly arise from such social learning processes. Of course, the cross-sectional design of this study can only confirm the existence of this association but cannot directly test the intermediate process of “observational learning.” Future research should incorporate longitudinal designs and specific measures of observational learning to further validate this mechanism.

Second, empathic concern has a significant indirect effect between childhood trauma and aggression. According to the General Aggression Model (GAM), input variables may be indirectly associated with behavioral outputs by influencing an individual’s internal affective state. Based on this GAM framework, the mediating effect of empathic concern can be explained through the following pathway: Childhood trauma may first weaken the individual’s affective internal state (i.e., empathic concern), making it difficult to generate normal caring responses to others’ suffering; this lower level of empathic concern is then associated with higher levels of aggressive output. Previous research has also shown a significant negative correlation between childhood trauma and empathy among individuals in compulsory drug rehabilitation (Ren et al., 2018), and studies comparing community and forensic samples have found that justice-involved individuals exhibit more pronounced empathy deficits than the general community population (Almeida et al., 2025).

It should be emphasized that the indirect effect of empathic concern was relatively small in magnitude (β = 0.076), accounting for only 25.25% of the total effect variance. This limited effect size suggests that aggression triggered by childhood trauma is likely a complex process involving multiple interacting factors. Synthesizing previous research, other psychological processes—such as emotional detachment (Kimonis et al., 2013) and callous-unemotional traits (Dackis et al., 2015)—may also constitute important parallel or alternative explanatory pathways. Future research should incorporate these variables simultaneously within a more integrative framework to more comprehensively elucidate the complex associations between childhood trauma and aggression. Furthermore, the above explanatory pathway is a theoretical inference based on cross-sectional data. The direction of the association between childhood trauma and empathic concern, and whether lower empathic concern actually leads to increased aggression, as well as their causal order and long-term effects, still need to be examined through longitudinal research.

However, we also noted certain heterogenous phenomena: the direct association between emotional neglect and aggression was not significant, whereas the other four dimensions of childhood trauma each showed a significant direct association with aggression. This difference suggests that the pattern of association between emotional neglect and aggression may differ from that of abuse-type trauma. Sexual abuse exhibited the strongest direct association with aggression, while emotional neglect showed the smallest direct effect. This heterogeneity may be related to the different characteristics of abuse and neglect: abuse is directly associated with individuals’ mental health and emotion regulation, and may be associated with more direct accumulation and expression of negative emotions; neglect, on the other hand, may be associated with stronger feelings of isolation and rejection (Dubowitz and Bennett, 2007). Our findings are consistent with the Dimensional Model of Adversity and Psychopathology (DMAP), which posits that abuse is typically perceived as a threat, whereas neglect is perceived as deprivation (McLaughlin and Sheridan, 2016). As a form of threat, abuse shows a stronger association with externalizing problems (e.g., aggressive behavior); neglect, as a form of deprivation, is more closely associated with internalizing problems (e.g., low self-esteem). This finding suggests that when assessing the consequences of childhood trauma, it is necessary to distinguish among different types of traumatic experiences, as they may differ in their pathways of association with individuals’ psychological and behavioral development.

#### 4.2.2 The mediating effect of perspective taking was not significant

This study found that the mediating role of perspective-taking between childhood trauma and aggression was not significant. This research indicates that the aggression driven by the negative input variable of childhood trauma may mainly be due to the imbalance in the emotional arousal pathway rather than the deficiency in the cognitive empathy pathway. On the path from childhood trauma to aggression, cognitive empathy (perspective-taking) and emotional empathy (empathic concern) may play different roles. The General Aggression Model suggests that input variables exert their effects by influencing three internal states: cognition, emotion, and arousal. Perspective-taking is mainly a cognitive internal state, that is, “knowing” what others are thinking and having a perspective. It is a neutral psychological ability. Aggression, especially in individuals who have suffered trauma, is often driven by intense emotions (such as anger, shame) and physiological arousal. Childhood trauma may weaken the individual’s affective empathy system, but may not impair the cognitive capacity for perspective-taking to the same extent. An individual with a history of childhood trauma may fully understand (perspective-taking) that their aggressive behavior will cause suffering to others, yet this cognitive understanding may not be accompanied by corresponding empathic concern; alternatively, their own anger and distress (emotional arousal) may be so intense that it dominates in conflict situations, preventing the cognitive understanding from forming an effective association with the inhibition of aggressive behavior. The non-significant mediating effect of perspective taking between childhood trauma and aggression may be related to the data collection method. For instance, because all data were self-reported, individuals’ assessments of their own perspective-taking ability may be inaccurate, particularly in cases of low insight or the presence of social desirability effects.

### 4.3 Implications for theory, research, and practice

First, this study examined the association between childhood trauma and aggression in incarcerated males, and tested the mediating effects of affective empathy (empathic concern) and cognitive empathy (perspective-taking). The findings delineate a pathway from adverse experiences (childhood trauma), through individual psychological characteristics (empathy), to an association with behavioral problems (aggression). The findings enrich the explanatory power of theoretical models such as social learning theory, the General Aggression Model, and attachment theory in accounting for the “cycle of violence” or the “intergenerational transmission of maltreatment.” This study may enhance understanding of the development of criminal psychology, and the indirect “early trauma–empathy–aggression” association it investigates provides new empirical evidence and theoretical perspectives for related fields.

Second, this study provides preliminary theoretical clues for the design of prison psychological correction interventions. When intervening with individuals who have a history of childhood trauma, merely enhancing their cognitive perspective-taking ability may be insufficient. The focus of intervention should be placed on both recalibrating their hostile attribution bias and improving their empathic capacity. Only when the individual’s “emotional” storm has calmed can the “cognitive” steering wheel regain direction. Empathy training may serve as one component of a comprehensive intervention; by training incarcerated individuals’ empathic concern ability, it may help compensate for the negative effects of early trauma and facilitate their resocialization. Although this study offers preliminary evidence for empathy-based interventions among incarcerated individuals, given the relatively small indirect effect size of empathic concern, we must avoid overstating the potential of empathy training alone to reduce aggression. A more effective intervention program would need to systematically integrate empathy training with multiple strategies such as anger management, impulse control, and cognitive reappraisal.

Third, regarding intervention implications, the interpretation of our findings should take into account the study’s limitations. In a sample of male incarcerated individuals who had been screened for severe mental disorders, the present study showed that empathic concern (rather than perspective taking) mediated the relationship between childhood trauma and aggression. This finding suggests that for incarcerated individuals with basic cognitive and emotional functions, intervention programs might attempt to cultivate empathic concern ability. However, for individuals with pronounced psychopathic traits (especially callous-unemotional traits), the fundamental prerequisites for developing empathy may be absent, and thus our conclusions should not be directly generalized to this subgroup. Future research, incorporating measures of psychopathic traits, is needed to further examine the applicability of empathic concern training across different subtypes of incarcerated individuals. Finally, it should be emphasized that all intervention implications drawn from this study are based on a preliminary investigation of cross-sectional associations; the findings provide only correlational evidence, and the effectiveness of any intervention remains to be validated by longitudinal studies.

### 4.4 Limitations and future recommendations

By constructing a mediation model, this study examined the association between childhood trauma and aggression as well as its mediating factors, which helps to identify psychological variables related to aggression among incarcerated individuals and provides a reference for optimizing future intervention programs. Several aspects of this study need further improvement:

First, this study employed a self-report method for data collection, which may be subject to social desirability bias. Future research could incorporate multiple measurement approaches, such as other-reports (e.g., evaluations by correctional officers) or experimental methods (e.g., empathy behavioral tasks). This study adopted a cross-sectional design, with all data collected at a single time point; therefore, it can only reveal associations among variables and cannot support causal inferences. Future studies could adopt longitudinal designs to obtain follow-up data, thereby clarifying causal relationships among variables and deepening our understanding of their dynamic associations.

Second, this study only examined the associations of childhood trauma and empathy with aggression. Future research should further incorporate other potentially relevant factors, such as personality traits of incarcerated individuals and parental modeling of empathy. In addition, childhood trauma may be differentially associated with different types of aggression (reactive aggression and proactive aggression). Future research should examine whether there are distinct pathways from childhood trauma to empathy and to different forms of aggressive behavior. A deeper understanding of the role that empathy plays in the association between childhood trauma and different types of aggression can provide insights and references for interventions targeting aggressive behavior among incarcerated individuals.

Third, this study has certain limitations in the coverage of demographic variables. Because variables such as sentence length, crime type, education level, and mental health history were not included during the questionnaire design phase, this study was unable to examine the potential confounding effects of these factors on the relationships among variables in the mediation model. Future research should systematically collect the aforementioned variables to more rigorously test the robustness of the findings. Although individuals diagnosed with severe mental illness by the prison medical service were excluded during participant screening, this study did not systematically assess psychopathic traits. Psychopathy is a complex clinical construct whose accurate evaluation typically requires specific diagnostic instruments, structured clinical interviews, and collateral file information, rather than being achievable through simple self-report questionnaires alone. Therefore, this study cannot completely rule out the potential confounding effect of psychopathic traits on the relationships among childhood trauma, empathy, and aggression. Future research that incorporates clinical assessment of psychopathic traits alongside questionnaire-based measurement will help to more precisely delineate the independence and boundary conditions of the relationships examined in this study.

Fourth, this study recruited only male incarcerated individuals from Inner Mongolia, which may limit the generalizability of the findings. Future research should expand the sampling scope and include female incarcerated individuals to more deeply explore the relationships among variables, thereby validating and extending the conclusions of this study. Additionally, this study did not include a matched community control group (i.e., individuals with a history of childhood trauma but without a criminal record). This design limitation makes it impossible to determine whether the association between childhood trauma and aggression, as well as the mediating effect of empathic concern, represents a pattern specific to the incarcerated population or is more generally prevalent among the broader population of childhood trauma survivors. Therefore, the conclusions of this study apply only to the specific population of male incarcerated individuals in Inner Mongolia, China, and should not be generalized to all individuals with a history of childhood trauma. Future research should include matched community samples to directly compare incarcerated individuals with non-criminal childhood trauma survivors on variables such as empathy and aggression, thereby achieving a more accurate understanding of the boundary conditions and population-specific characteristics of the relationship between childhood trauma and aggression. Finally, it must be emphasized that the pathway identified in this study is only one of many possible developmental trajectories following trauma, and should not be misinterpreted as implying that childhood trauma inevitably leads to aggressive behavior or criminal outcomes.

## 5 Conclusion

(1). Childhood trauma is positively associated with aggression among male incarcerated individuals.
(2). Empathic concern has a significant mediating effect between childhood trauma and aggression.
(3). The mediating effect of perspective taking between childhood trauma and aggression is not significant.

## Data Availability

The raw data supporting the conclusions of this article will be made available by the authors, without undue reservation.
